# Gut Fermentation of Dietary Fibres: Physico-Chemistry of Plant Cell Walls and Implications for Health

**DOI:** 10.3390/ijms18102203

**Published:** 2017-10-20

**Authors:** Barbara A. Williams, Lucas J. Grant, Michael J. Gidley, Deirdre Mikkelsen

**Affiliations:** ARC Centre of Excellence for Plant Cell Walls, Centre for Nutrition and Food Sciences, Queensland Alliance for Agriculture and Food Innovation, The University of Queensland, St. Lucia QLD 4072, Australia; l.grant3@uq.edu.au (L.J.G.); m.gidley@uq.edu.auF (M.J.G.); d.mikkelsen@uq.edu.au (D.M.)

**Keywords:** large intestinal fermentation, microbiota, polyphenols, plant cell walls, fruit, vegetables, cereals, short-chain fatty acids

## Abstract

The majority of dietary fibre (DF) originates from plant cell walls. Chemically, DF mostly comprise carbohydrate polymers, which resist hydrolysis by digestive enzymes in the mammalian small intestine, but can be fermented by large intestinal bacteria. One of the main benefits of DF relate to its fermentability, which affects microbial diversity and function within the gastro-intestinal tract (GIT), as well as the by-products of the fermentation process. Much work examining DF tends to focus on various purified ingredients, which have been extracted from plants. Increasingly, the validity of this is being questioned in terms of human nutrition, as there is evidence to suggest that it is the actual complexity of DF which affects the complexity of the GIT microbiota. Here, we review the literature comparing results of fermentation of purified DF substrates, with whole plant foods. There are strong indications that the more complex and varied the diet (and its ingredients), the more complex and varied the GIT microbiota is likely to be. Therefore, it is proposed that as the DF fermentability resulting from this complex microbial population has such profound effects on human health in relation to diet, it would be appropriate to include DF fermentability in its characterization—a functional approach of immediate relevance to nutrition.

## 1. Introduction

Today, in affluent societies, there is a well-publicised epidemic of obesity, along with related chronic diseases such as type 2 diabetes, cardiovascular disease, and cancer, particularly of the large intestine (LI) [1,2]. Many epidemiological studies [3,4,5] have indicated a strong link between low levels of dietary fibre (DF) and the incidence of these diseases. DF, originating from fruits, vegetables and whole grains, have been shown to have very specific positive health benefits including: stabilisation of blood glucose concentrations [6], laxation [7], and cholesterol attenuation [8]. They have also been associated with a reduction in gastro-intestinal tract (GIT) disorders such as Crohn’s disease, and ulcerative colitis [9,10,11]. Hence, the current interest in DF as an essential part of a healthy diet. However, given this interest, it is noteworthy that within nutritional guidelines, “dietary fibre” is often considered as a single entity (although sometimes classified as soluble vs insoluble), though it is now known to be more complex and includes a wide range of different compounds, which vary substantially in their biological and chemical properties. These compounds can range from cellulosic materials, to resistant starch, to non-digestible oligosaccharides.

DF represents the major non-digestible component in most diets and exerts a physiological influence throughout the digestive tract through structuring of digesta (relevant to e.g., satiety and control of food intake), modulation of digestion processes (relevant to e.g., control of circulating glucose and lipid levels), and acting as a prime substrate for microbial fermentation (relevant to control of laxation and reduced risk of colon cancer amongst other chronic diseases). The emphasis here is on the interactions with microbiota.

In this review, we examine what is known about different forms of DF, by an analysis of specific components of plant cell walls (PCW). This will be accompanied by what is known about how the GIT microbiota responds to these compounds. In addition, information comparing these purified materials with whole foods will be included. Lastly, recommendations will be made as to how “dietary fibre” could be classified differently, in relation to its fermentability, rather than only its solubility.

## 2. Plant Cell Walls and Their Components-Definition and Physico-Chemical Properties

The majority of DF originates from PCW which are key in maintaining plant structure and function. Chemically, DF mostly comprise carbohydrate polymers, which resist hydrolysis by mammalian digestive enzymes in the small intestine, but can be fermented by bacteria, mainly in the LI [12,13]. In terms of the functional properties of DF for humans, its health benefits have been attributed to a combination of rheological and biophysical behaviours, its function as a matrix, and its biochemical traits. The main benefits associated with DF fermentability relate to its effects on microbial diversity and function within the GIT, and the associated by-products of the fermentation process [10,14].

Various classification systems for DF exist, and in large part relate to the requirements of a range of different professional groups including dieticians [15], and animal nutritionists [16,17,18]. The most common physico-chemical classification of DF for human nutrition purposes is to divide it into two sub- groups based on its solubility in water, as an indicator of its “potential” functionality and physiological effects in monogastrics [19]. In terms of behaviour in the GIT, water solubility is considered to be a useful predictor of its water-holding capacity, viscosity, and degree of fermentation by GIT bacteria [20]. For example, the degree to which PCW polysaccharides can be fermented varies considerably, with lignin (considered to be insoluble) being very resistant to fermentation, and pectin (highly soluble), usually being fermented to completion. Generally, it has been assumed that soluble fibres are fermented more rapidly compared with insoluble fibres [21] though this view is changing [22,23]. It should be noted, however, that there is no standardised method for separating soluble and insoluble fibres, and conditions used may vary in terms of temperature (usually close to physiological), water or buffer as solvent, and fibre to solvent ratio. All of these can influence the partition of fibre materials into soluble and insoluble fractions, so the categorisation has significant limitations.

### 2.1. “Soluble” Dietary Fibres

**Figure 1:**
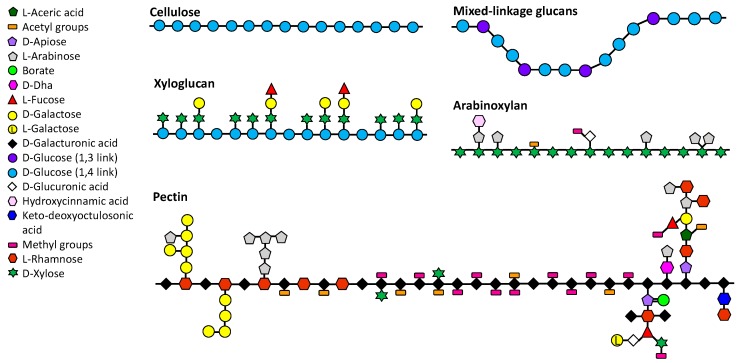
Schematic depiction of key soluble and insoluble dietary fibre structures which form the chemical components comprising the plant cell wall. The backbone structures for cellulose, mixed-linkage glucans, xyloglucan and arabinoxylan are (1,4)-β-linked, while the backbone of pectin is comprised of (1,4)-α-linked chains of galacturonosyl residues. In the pectin structure, the left hand part containing alternating rhamnose and galacturonic acid in the backbone is rhamnogalacturonan I, the middle section without long branches is homogalacturonan, and the right hand section with complex multi-sugar branches is rhamnogalacturonan II. Chain aggregation is prevented for xyloglucan, arabinoxylan and pectic non-cellulosic wall polysaccharides, due to the presence of short oligosaccharide-, monosaccharide- or acetyl group side chains. For mixed-linkage glucans, on the other hand, it is the irregular conformation of this polysaccharide which prevents main chain aggregation (Adapted from Burton et al., 2010 [50]).

Soluble DF can increase the viscosity of digesta depending on its chemical structure, and molecular weight which affect the conformation of these polymers in solution. This in turn, can lead to a reduced glycaemic response [14,19], by delaying gastric emptying and nutrient release as well as by inhibiting the action of α-amylase [24], thus regulating blood glucose [25]—a critical mechanism related to the development of insulin resistance and then Type 2 diabetes [26]. So-called soluble fibres are found as part of most PCW, though vary in their structure, molecular size, and also, interestingly, in their solubility. Figure 1 shows the chemical structure of several specific soluble and insoluble fibres.

#### 2.1.1. Pectin

“Pectin” refers to a group of covalently-linked polysaccharides, structurally consisting of ~70% galacturonic acid [27], characteristic of the cell walls of most plants, including fruits and vegetables. The main homogalacturonan backbone of pectin comprises α (1–4) linked d-galacturonic acid residues. Many of these acids are in the form of methyl esters. This degree of esterification (DE) can, in principle, range from 0 to 100%, but in plants is normally 50–80%, and is important in determining physical properties [28]. Therefore, pectin is usually classified as high methoxyl DE > 50% or low methoxyl DE < 50%. A high DE pectin can form gels at pH < 4.0 or at high concentrations of soluble solids, such as sucrose. A low DE pectin can form stabilised gels with metal cations such as calcium [29]. Homogalacturonan is the most abundant type, comprising approximately 65% pectic polysaccharides found in fruits and vegetables [27]. The other two main types are: rhamnogalacturonan-I (RG-I) which contains a backbone of alternating galacturonic acid and rhamnose with oligosaccharide side chains or arabinose and/or galactose attached to some rhamnose residues, and rhamnogalacturonan-II (RG-II), which is a complex small polymer containing many different sugar residues [30]. Recent work [31] detailing the deconstruction of RG-II side chains by bacterial enzymes, has shown that the current structural model of RG-II may have to be revised.

#### 2.1.2. Arabinoxylan

Arabinoxylan (AX) belongs to a class of heteropolymers called hemicelluloses, a major polysaccharide component of PCW in cereals such as wheat and rye. In general, AX has a backbone of 1,4 linked β-d-xylose residues with α-l-arabinose residues attached as single side-chains to positions 2 and/or 3 of xylose. The ratio of arabinose to xylose can be used to describe a characteristic of AX structure. AX accounts for approximately 20% of the content of wheat bran [32], and, in its purified form has been shown to be readily fermentable both in vitro [33], and in the caecum of grower pigs [34]. In addition, it was shown that for different AX-containing rye milling fractions, there were significant differences in fermentability, which were ascribed to variation in alkali-labile crosslinks with lignin, rather than actual AX structure [35]. Recently, it has been shown that there is a highly complex xylan-degrading apparatus within the large intestinal microbiota which is fine-tuned to recognize different forms of complex carbohydrates, and respond accordingly [36]. Related glucuronoarabinoxylans are characteristic of maize and sorghum.

#### 2.1.3. Mixed-Linkage Glucans

Mixed-linkage glucans (MLG) are polysaccharides present in varying proportions within the PCW of cereal grains, being a major component in oats, rye, and barley, and to a lesser extent in wheat. MLG is an unbranched linear polymer of β-(1,4) glucosyl residues interrupted by β-(1,3) residues incorporated into the chain typically at a ratio of between 1:3 or 1:4. These long chains of irregular molecular structure increase water solubility and gel-forming properties [37]. In addition, they have also been demonstrated to have a range of health-related properties, including hypoglycaemic effects [38,39], and a reduction in circulating bile acids and cholesterol [40]. MLG has also been shown to be fermented both in vitro [41,42], and in vivo in rats [43], pigs [44,45,46] and human subjects [47].

#### 2.1.4. Xyloglucans

Xyloglucan is typically the most abundant hemicellulose in primary cell walls of most dicots and non-graminaceous monocots (i.e., fruits, vegetables and other vascular plants). It comprises a backbone of β1 → 4-linked glucose residues, most of which are substituted with α 1 → 6 linked xylose sidechains. Xyloglucan can bind to the surface of cellulose microfibrils, becoming incorporated into the cell wall network [48]. Little work has been reported linking xyloglucan with specific health benefits, though its ubiquity in the human diet suggests that more work may reveal important connections with human health. One group has shown that its metabolism may be mediated by a niche species *(Bacteroides ovatus*) which has implications for gut microbial ecology, and therefore health [49].

#### 2.1.5. Others

Although the above sections cover the major potentially soluble fibre polymers present in plant cell walls, there are others that are used as food additives which are derived from specialised cell walls (e.g., guar galactomannan and konjac glucomannan), algal cell walls (e.g., alginate, carrageenan, and agar), or plant energy reserves (e.g., inulin, fructo-oligosaccharides, and galacto-oligosaccharides). These are outside the scope of the current review, but are expected to have generally similar properties to soluble fibre polymers from typical plant cell walls.

### 2.2. “Insoluble” Dietary Fibres

For GIT bacteria, insoluble fibre poses a significant challenge due to its reduced accessible surface area [51], and the hydrogen-bonding networks which hold the carbohydrate chains together [51].

#### 2.2.1. Cellulose

Cellulose is the most abundant organic polymer on earth [52]. Consisting of 1,4 linked β-glucosyl residues, cellulose chains are synthesised at the cell-surface directly into the PCW and rapidly associate into semi-crystalline microfibrils of probably 18 or 24 chains. These may then further aggregate into a characteristic fibre ribbon [53,54]. The combination of cellulose, cross-linking hemicelluloses and interpenetrating pectins provides strength and rigidity to the PCW, which make it highly resistant to mechanical and chemical degradation [55]. Cellulose is generally considered to be soluble only in strong alkali solution (4–6 M), but there is a portion (amorphous) which is more readily hydrolysable in acid [56].

While considerable work has been done in this field in ruminants, as already reviewed exhaustively [57,58], the extent to which cellulose influences the monogastric GIT microbiota and vice versa, is currently less well understood [59].

#### 2.2.2. Lignin

Lignin, a complex polymeric network of phenolic compounds, is commonly found as part of secondary cell walls, specifically of woody tissues [60]. It is a structural component, with the proportion of lignified cell walls increasing with maturation [19]. While a complex polymer, it is not a polysaccharide, and contains ~40 different oxygenated phenyl-propane units. It is considered to be chemically inert [56]. Lignin is embedded in the cell wall between cellulose and hemicelluloses, with varying degrees of concentration depending on plant species, stage of maturation, and cell type. It is synthesised during secondary cell wall formation and is distributed throughout the wall [61]. However, it is minimally consumed, with consumption estimated at <1 g per day for humans [62], and so will not be considered further in this review.

### 2.3. Limitations of Classification by Polymer Type

Although individual fibre polysaccharides can be defined and quantified based on their distinctive chemical compositions, they are rarely consumed as purified polymers. The most common form of DF consumed is as PCW from cereals, fruits, vegetables and other plant-based foods. Here, “soluble” polysaccharides are typically present alongside insoluble cellulose in a hydrated but insoluble form. This has two major consequences for their functional classification. On the one hand, classification of food PCWs as insoluble implies a functional equivalence to cellulose, whereas the high water-holding capacity and rheological properties have more in common with soluble polymers. On the other hand, a chemical classification would identify components such as pectins and arabinoxylans as soluble, whereas in reality, they are often present in foods as part of insoluble plant cell walls. There is clearly a need for a more functional classification of fibre types as found in food to overcome these limitations.

### 2.4. Phytonutrients from Plants

**Figure 2:**
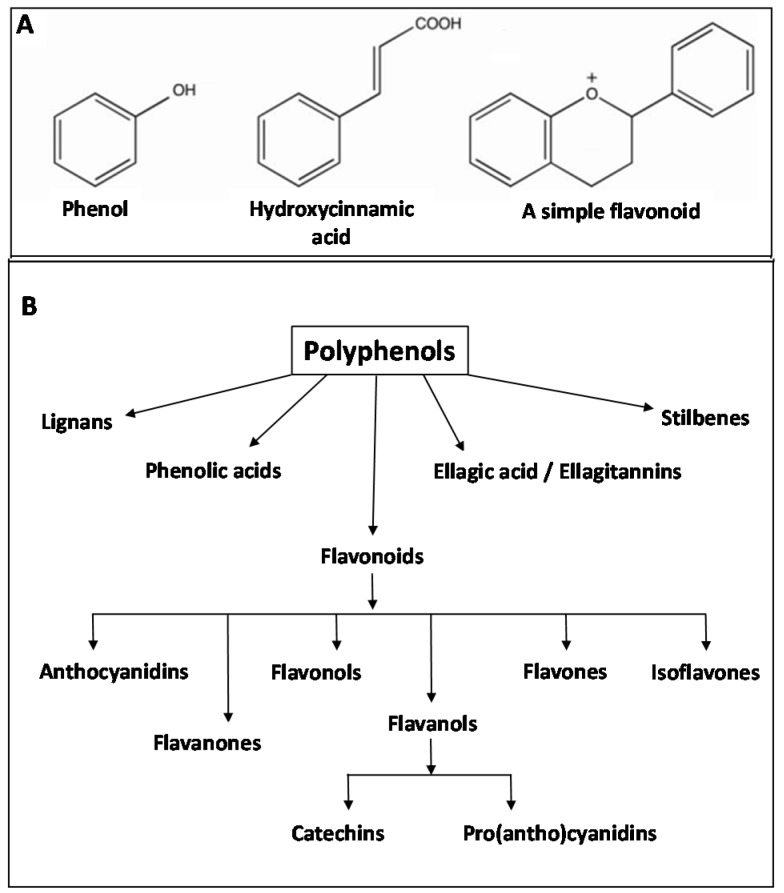
Basic structure of (**A**). Some of the simplest phenols and flavonoids (adapted from Khoddami, Wilkes et al., 2013 [68]), and (**B**). The common classes of polyphenols found in fruits and vegetables (adapted from Singh et al., 2011 [69]).

Some nutritional benefits of plant-based foods have also been partly attributed to phytochemicals, which are secondary metabolites such as polyphenolic compounds and carotenoids, abundant in fruits, vegetables and grains [63]. Soluble polyphenols accumulate within vacuoles and can become attached to PCWs after processing into food and/or in the digestive tract, while some simple flavonoids and ferulic acid esters are actually incorporated into the cell wall structure [64]. Polyphenols are a group of heterogeneous compounds consisting of hydroxylated phenyl moieties. They comprise multiple simple phenols or phenolic acids, with differing numbers of phenolic rings and substituting groups and are classified into two groups: the flavonoids and the non-flavonoids [65,66,67]. Flavonoids are the major group with over 9000 unique structures found in nature. Figure 2 shows an example of the basic structure of the simplest phenols and flavonoids (A) and the most common classes found in fruits and vegetables (B).

Once consumed, the more complex polyphenols may have poor bioavailability, compared with other macro- and micronutrients [67,70]. In part, this may be because they are bound to the PCW, which will also mean that absorption cannot occur until released [71,72]. Estimations put polyphenol absorption in the small intestine at 5–10% and the remaining 90–95% accumulates in the large intestinal lumen up to millimoles in concentration, where they may be subjected to microbial fermentation [73,74].

While not classified as DF, phytochemicals and DF have an intricate relation, and so need to be examined together as part of relevant food sources.

### 2.5. Effects of Food Processing on Plant Cell Walls

Until recently, dietary advice has focused on nutrient content of foods according to their individual macro- and micro-nutrient contents, including DF as a single nutritional category [75]. However, the issue of food complexity, and how that complexity can change with food processing has been only rarely taken into account [76]. Therefore, it is being realized that more studies should compare whole food structures and their complex components, under the influence of various forms of food processing. For example, this has been strongly recommended for the study of whole grains [77], and fruits and vegetables [4]. In addition, Liu pointed out that the consumption of the complex mixture of phytochemicals in whole fruits and vegetables was likely to be of greater benefit than the single phytochemicals in isolation [78].

In addition, definitions have been put forward recently to categorize plant foods as whole, processed, and ultra-processed [76]. The main difference between these groups is centred on their level of complexity, in terms of increasing digestibility, the variety of different chemical structures within those foods and how they are bound to each other. The proposed definition categorises whole plant foods as those which have been minimally processed, and include fruits, vegetables and whole grains. Processed foods, on the other hand, are proposed to be those ingredients derived from whole foods, such as cooked foods, or oils, flour, starches, and sugar, which have been derived from the whole foods in some way. The proposed definition of the ultra-purified group is that it comprises those foodstuffs which are made up using ingredients of the processed food group. Largely, it is DF and phytochemical-rich foods which are absent from these ultra-processed foods, and it is these constituents which are being seen as an essential component for overall host health, and of the gastrointestinal tract (GIT) microbiota [75,76].

## 3. Gastro-Intestinal Tract Fermentation and Effects on Health

For many years, it has been recognised that a significant proportion of the health benefits of DF result from the presence and activities of the host’s resident microbial population within the GIT [11]. These microbes are responsible for breaking down cell wall polymers, leading to the production of short chain fatty acids (SCFA), which are known to have important roles in terms of host health, including homeostasis of the GIT [79]. Given the dual complexity of the GIT microbiota and plant cell walls, research linking identity and fermentative activity of microbes during DF fermentation has previously been limited. However, it is now a major area of investigation on the back of the cheap and accurate molecular characterisation of microbial species which is now possible using DNA sequencing techniques [49,80,81] coupled with detailed enzymology studies.

### 3.1. Gastro-Intestinal Tract Microbiota

The GIT microbiota encompasses the microbial population within the entire GIT, from the mouth to the anus. It includes bacteria, fungi, viruses and archaea, though the bacteria are in the overwhelming majority [82]. In monogastrics, the predominant site of fermentation is the LI [83], though it is now being recognized that some fermentation can also occur within the stomach and small intestine, particularly at the terminal ileum [84].

These resident bacteria play an essential role in normal digestive function, immune development, brain development and pathogenic defence [85,86]. Although the intricate details of the role bacteria play is not fully understood, it is now commonly referred to (collectively) as the “other” human genome [87]. The microbiota is a partially stable ecosystem, and GIT bacteria have the ability to resist significant challenges presented by their dynamic environment, enabled by the broad metabolic potential of their genes [88]. This is particularly true of a population with high diversity of microbial species [89].

The human GIT bacterial community has been classified into at least seven phyla, of which four are the most predominant (usually ~98% of the total population). These are the Firmicutes (58–88%), Bacteroidetes (8.5–28%), Proteobacteria (0.1–8%), and Actinobacteria (2.5–5%) [90,91,92]. Bacterial community profiling from faeces has shown that as many as 60% of bacterial species have not yet been identified [11,93]. For this reason, many studies report at higher taxonomy than the level of species, due to the lack of representative sequences in prokaryote databases [94,95]. A recent review [96], provides an excellent summary of how the human microbiome can regulate and maintain human health.

The human GIT microbiota is still a relatively new frontier for studies using next-generation sequencing, and new species are constantly being sequenced and added to public databases. In the past, many studies have focused on the impact of disease and significant “detrimental” bacteria, such as the role of *Helicobacter pylori* in stomach ulcers [97,98] and *Escherichia coli* in diarrhoeal and various extra-intestinal diseases [99]. In addition, there is much research which attempts to elucidate the role of bacteria in the development of obesity, Crohn’s disease, irritable bowel syndrome [97,100,101,102], and other chronic diseases.

GIT bacteria have evolved with their hosts to be symbiotic. In general, to avoid competitive pressures with each other, there is an organised trophic structure, a type of “food chain” [103], by which species have specific roles to play, though these may be interchangeable to some extent. In terms of influencing our GIT microbiota, the diet is considered to be one of the easiest ways of impacting the microbial population [104]. However, it is also currently the least defined and most elusive to comprehend in the scientific literature [105,106,107], in part because of the complexity of diets required to maintain human/animal health.

### 3.2. Microbial Function

As a whole, the bacterial population is extremely competitive and diverse, with much of its energy-obtaining metabolic activity being saccharolytic [108]. However, the GIT bacteria are not dependent on simple sugar availability, and can obtain energy and carbon sources from carbohydrates of a more complex nature [109] such as found in DF. Complex polymers are broken down by a suite of bacterial enzymes such as polysaccharidases, glycosidases, proteases and peptidases [110]. These enzymes degrade polymers (polysaccharides, proteins) into their respective sugar or amino acid components. Bacteria can then ferment these smaller components into SCFA and other carboxylic acids, CO_2_, H_2_, and other end-products such as ammonia and branched-chain fatty acids (BCFA) [111], and those of various phytonutrients. This process can involve so-called “cross-feeding”, whereby several species act together on a complex molecule to complete the process [109]. Given the complexity and numbers of species involved, the overall metabolism of these bacteria in the GIT is also very complex, and assessments of human and animal GIT health outcomes are often analysed by a number of indicators and parameters for GIT health. These include SCFA and NH_3_ production, digesta transit time, pH, and stool quality [112,113,114].

#### 3.2.1. Low Dietary Fibre Diets and Pathogenesis Associated with Microbiota

Many recent studies have emphasized the proliferation of proposed probiotic bacteria such as species of the *Bifidobacterium*, *Eubacterium* and *Prevotella* genera. These bacterial groups have been targeted in an effort to essentially “out-compete” other potentially pathogenic genera such as the Family Clostridiaceae [115,116]. For example, a DF-deficient diet, led to a dramatic increase in bacterial populations degrading host-secreted mucus glycoproteins in a murine model [117], promoting an aggressive colitis by an enteric pathogen. This indicated the importance of a constant supply of carbohydrates for GIT bacteria. Reduced bacterial diversity is considered an essential aspect of bacterial dysbiosis and has been associated with an increased incidence of colo-rectal cancers [118].

#### 3.2.2. Diet and microbiota Stability

It is becoming clear that a healthy gut microbiota is one which exhibits diversity, stability and resistance. More evidence is accumulating that a more complex diet, containing a wide range of DF structures and molecules, is associated with increased diversity of the faecal population of monogastrics such as pigs [119] and humans [36,81]. Stability over a longer period of time, is an indicator of overall microbiota health. Resistance is also a key characteristic, particularly at times of significant lifestyle changes, such as weaning of infants, antibiotic treatment, and recovery from some illnesses when the microbiota can shift quite dramatically. However, it appears that by maintaining or introducing a diet rich in DF, stabilization can occur faster.

In a study by Castillo et al. [120] using pigs, the time taken for the microbiota to change and stabilize in response to a change in diet was reported to be up to six weeks within the caecum and proximal colon [120]. However, samples were taken on Days 0, 7, 21 and 42, though the population may have stabilized earlier than Day 42, given the 21 days between the final two samples. Within a 16-day period, on the other hand, Gorham et al. [119] found that stabilisation of the GIT microbiota varied according to the DF content of the diet, with higher DF leading to a generally more rapid stabilisation by Days 9–16 for pigs fed either a diet containing whole wheat or β-glucan. However, by Day 16, the microbiota was still not completely stable.

#### 3.2.3. Diet and Microbial Diversity

A diverse microbiota containing a wide range of potential functions, has been identified as an important aspect of a healthy GIT microbiota [121]. At least in part, it is likely that this is because a larger number of bacterial species will have a much larger gene pool which are then able to fulfil a wider variety of functions, leading to a microbiota which is more stable against potential perturbation [122]. It is becoming clear that consumption of a wider variety of different and more complex dietary compounds (such as polyphenols, carotenoids, and various DF including PCW), consumed as whole foods with increased complexity, appears to be related to greater bacterial diversity [123]. In a study of American microbiomes from individuals consuming a generally Western diet, the bacterial populations had enhanced numbers of genes associated with the degradation of amino acids and simple sugars [124], while other studies have found increased production of beneficial SCFA and the potential for butyrate production to be higher in non-Western microbiomes [125,126].

In terms of effects on health, Salonen and De Vos concluded in their review, that reduced bacterial diversity was associated with obesity [127]. This finding was supported by several studies whereby a high fat diet was shown to reduce intestinal bacterial diversity in mice [123], though whether this is due to the presence of fat itself, or the stimulation of bile acid production is still unclear.

### 3.3. Fermentation End-Products Including Short-Chain Fatty Acids, Ammonia and Others

The monogastric GIT fermentation process results in a number of end-products which may or may not be beneficial for the mammalian host. Broadly, gut fermentation can be divided into two categories, the fermentation of carbohydrates and the fermentation of proteins.

The by-products of bacterial fermentation have been shown to have both beneficial and detrimental health effects. The metabolites of carbohydrate breakdown are SCFA, primarily acetate, propionate and butyrate, and the synthesis of these products has been shown to have benefits including: (i) reduced inflammation in IBD [128]; (ii) an energy source for colonic mucosa cells; and (iii) differentiation and apoptosis of host colonic cancer cells [129]. Propionate and acetate can also function as substrates for lipogenesis and gluconeogenesis in the liver and peripheral organs [130]. This is in contrast with those by-products of protein metabolism, which apart from branched-chain SCFA also include amines, phenols, thiols, dihydrogen sulphide and ammonia [131]. If beneficial saccharolytic bacteria are present, these by-products can be utilised during cellular processes [131]. Otherwise, the build-up of these products can have adverse effects on human health, and have been implicated with increased risk of colorectal cancer and ulcerative colitis [132].

#### 3.3.1. Carbohydrate Fermentation

Bacterial fermentation of carbohydrates results predominantly in the production of SCFA such as acetic, propionic and butyric acids, but a range of other carboxylic acids can also be produced, including lactic acid [83]. These end-products of bacterial fermentation of carbohydrates are generally beneficial for GIT health [133,134]. Once produced, the SCFA can have multiple effects within humans (as summarised in Table 1) and other mammals. They are heavily utilised as a source of energy, by both humans [135] and bacteria [136].

Acetic, propionic and butyric acid consist of 2, 3 and 4 carbon atoms respectively, and are the principal products of carbohydrate fermentation by bacteria in the GIT [138]. SCFA production is a process involving a range of reactions and metabolic processes during the anaerobic breakdown of organic material by bacteria. The SCFA usually occur in proportions of acetate > propionate > butyrate at approximate output levels of 60, 25, and 15 percent respectively [83,138]. Within the LI, SCFA are important promoters of colonic health as they are implicated in controlling colonic mobility, colonic blood flow and GIT pH, all of which has an effect on nutrient and electrolyte absorption [83,141].

The SCFA, as well as lactic and succinic acids, also play an important role in the cross-feeding of intermediary metabolites by the gut bacteria. Depending on the individual species of bacteria, they may be involved as essential growth requirements, or lead to changes in the GIT environment such as reduction of pH, or activity as either growth promotors or inhibitors [140,142]. The re-utilization of partial breakdown products from fermentation [142] is also important in terms of providing a rich range of substrates for a wider variety of bacterial species. For example, it has been suggested that as the Bacteroidetes encode a large variety of polysaccharide-degrading enzymes, and so carry out the primary degradation of polysaccharides, the Firmicutes utilizing a wide variety of smaller sugars, then thrive as cross-feeders within that environment [51], again suggesting that a wider variety of molecules of varying size and structure can support a wider variety of bacterial species. The subject of bacterial cross-feeding in the GIT has been extensively reviewed [51,143].

Acetic acid is the predominant SCFA in venous blood [141]. Acetic acid produced in the LI is absorbed across the GIT epithelium wall into the portal vein, and diffuses through the peripheral venous system [83]. In the body, acetic acid can induce apoptosis of cancerous cells [144] and prevents DNA oxidative damage caused by hydrogen peroxide in epithelial cells of the distal colon [145]. Acetic acid has been shown to be the principal SCFA fermentation product of pectin and xylan in the GIT [146].

Although propionic acid can be metabolised from a range of substrates, including proteins, the most common metabolic pathway involves fermenting carbohydrates [147]. Propionic acid is absorbed into the portal vein and moves to the liver where it can be metabolised by hepatocytes [83]. Approximately 90% of propionic acid absorbed into the portal vein is metabolised in the liver, of which a substantial proportion is used for gluconeogenesis [138].

Butyric acid is a major oxidative fuel for colonocytes (colonic epithelial cells), supplying approximately 60–70% of their energy requirements [135]. Associated with this function, it has been thought that butyric acid will have important properties in the prevention of colonic cancer [148,149]. Butyric acid ingested in the diet is fully absorbed in the small intestine, so the only source available in the LI is as the product of fermentation [138].

Lactic acid can be the metabolic end-product from a range of mono- and poly-saccharides in the human diet, including some common substrates such as sucrose, lactose, starch, glucose, or xylose [150]. Lactic acid is one of the most commonly produced carboxylic acids in the GIT, and many bacteria have the metabolic pathways for its synthesis [151]. However, it is mainly found in the small intestine where there is a less diverse and abundant microbial population, but rarely in the LI where the highly complex and abundant microbial population can utilize it rapidly [152].

#### 3.3.2. Protein Fermentation

Protein fermentation refers to the breakdown of amino acids by bacteria, and normally increases when there is a shortage of energy available in the form of fermentable carbohydrates. Health benefits of reduced protein fermentation are related to the reduction of ammonia and other nitrogenous compounds in the GIT [153], while increased protein fermentation is considered to be detrimental to GIT health [112].

Ammonia (NH_3_) is the dominant by-product of the fermentation of amino acids in the GIT. Excess protein fermentation can lead to an increase of NH_3_ and amines. NH_3_ then moves from the GIT into the bloodstream and is detoxified in the liver or muscles, with a large amount converted to urea and excreted by the kidneys [154].

Protein fermentation can also result in the production of branched-chain SCFA, amines, phenols, sulphides and thiols [131]. With the exception of branched-chain fatty acids, an excess of these metabolites has been linked to colorectal cancers, ulcerative colitis and other severe bowel disorders [132,155]. However, if there is a constant supply of carbohydrates and sufficient saccharolytic bacteria, the detrimental effects of these metabolites can be significantly reduced [131].

#### 3.3.3. Fermentation of Polyphenolic Compounds

Increasingly, it has been shown that certain polyphenols, ingested as part of the diet, have beneficial health properties [67,156]. For example, it has long been thought that their anti-oxidative properties would be beneficial [67]. However, it is only more recently, that it has been realized that bioavailability is an important factor which influences the physiological properties of these chemicals; in other words, whether or not they can be taken up across the GIT mucosa and into the bloodstream [157]. Bioavailability is when the polyphenols are available in the target tissue to exert their effect. The bioavailability of polyphenols is strongly influenced by the biotransformation undertaken in the GIT by microbial populations [158]. However, bioaccessibility may delay this process in whole foods. Bioaccessibility is the amount of specific constituents (i.e., polyphenols) released from the food matrix. Only after polyphenols are released from the food matrix by solubilisation, digestive enzymes or bacterial fermentation do they become potentially bioavailable [159]. DF has an important role to play here, as many polyphenols can bind to PCW components [72] and, once bound, are relatively resistant to release under the conditions [160] found in the stomach and small intestine. This results in the predicted passage of a significant proportion of dietary polyphenols to the LI where they are available to interact with the resident microbiota.

In general, the interaction between phytonutrients and the GIT microbiota can be split into two broad categories. Either the GIT bacteria degrade larger molecules to smaller ones, which may then be absorbed across the intestinal mucosa. Conversely, it is also possible for certain phytonutrients to have antimicrobial effects on specific microbial species within the GIT. In addition, both mechanisms may occur simultaneously [161]. In addition, in a review [162], some details were given of the bioconversion of lignans, though this was not considered to occur to a great extent in the GIT. In another study, the bioavailability of purified flavonoids was investigated [163] specifically, using an in vitro model of the pig caecum, to examine the microbial deconjugation and degradation of the most common flavan-3-ols. The flavan-3-ols used were mostly metabolised by the GIT microbiota within 4–8 h to monomeric flavonoids and hydroxylated phenol-carboxylic acids, which were speculated to be responsible for antioxidant activities [163].

Sanchez-Patan and co-workers [164], assessed the metabolism of phenolic compounds comparing a cranberry and a grape seed extract by the GIT microbiota, using an in vitro model of the ascending and descending colon, without previous intestinal digestion. This confirmed the formation of new bioavailable compounds by the action of inoculated microbiota on the cranberry extract. Under the same conditions, the grape seed extract polyphenols were metabolised to a lesser extent because of an observed antimicrobial effect specifically against *Bacteroides*, *Prevotella*, *Blautia coccoides*, and *Eubacterium rectale*. In a separate study feeding catechin to rats, it was found that the presence of catechin was associated with an alteration in the composition of the gut microbial population as well as a down-regulation of the diversity of the rats’ gut microbiota [165]. The consumption of high-flavonoid whole foods also supported these findings of antimicrobial effects of polyphenols, with a significant decrease in *Clostridium leptum*, *Ruminococcus bromii*/*flavefacians* in the high flavonoid whole-foods diet cohort [166].

## 4. Comparing Fermentation of Purified Dietary Fibre (DF) and Whole Plant Foods

In recent years, many studies have highlighted the negative effects of high fat and/or low fibre in the diet in relation to the GIT microbiota (including decreased diversity) [167,168]. In addition, more research is showing that there is a positive relationship between a variety of DF and the GIT microbiota [169]. A selection of these is shown in Table 2. There are fewer studies which examine extracts of (mainly) fruits in terms of their impact on the GIT microbial populations [164,170]. However, there are very few studies which have examined the impact of whole foods, particularly fruits and vegetables, in terms of how they could potentially “improve” microbial diversity and stability.

Consequently, it is increasingly of interest to analyse the fermentation properties of both single extracted components of PCW, but also the PCW within an actual plant food, particularly after various levels of processing. In addition, there is a body of work using bacterial cellulose composites as a model for plant cell walls [171]. In vitro fermentation of these composites allows the examination of “simplified” plant cell walls to elucidate mechanisms of microbial activity in response to model cell walls [172].

### 4.1. Purified Compounds Affecting GIT Microbiota

Many studies have focused on the testing of a single purified source of DF, usually one extracted from plants, sometimes by the use of harsh chemical procedures. Some of these have been used later as food additives known as “prebiotics”. The original definition of a prebiotic first coined in 1995 [173] was: “Prebiotics are non-digestible food ingredients that beneficially affect the host by selectively stimulating the growth and/or activity of one or a limited number of bacterial species already resident in the colon, and thus attempt to improve host health”.

This definition included compounds such as inulin, resistant starch and various oligosaccharides [105,174]. At that time, a prebiotic was generally to be a single (usually a comparatively simple carbohydrate) ingredient that would be fermentable in the GIT, leading to a positive shift in the microbiota, and therefore considered to support GIT health [175].

However, since then, this definition has been modified several times, as summarized by Bindels et al. [176]. More importantly, these authors propose a fundamental change to the original definition, by proposing that the original definition be modified as follows [176]: “…a prebiotic is a nondigestible compound that, through its metabolization by microorganisms in the gut, modulates composition and/or activity of the gut microbiota, thus conferring a beneficial physiological effect on the host”.

In other words, the most important change is that it is alterations in GIT bacterial metabolism, rather than specific numbers of bacterial species which receives the most attention. Interestingly, this widens the possibilities in terms of potential prebiotics, potentially including more complex or whole foods, versus the more purified compounds of recent years.

### 4.2. “Purified” Dietary Fibre

Table 2 shows some examples of purified components which have been used as ingredients to test their effect on the faecal microbial community and function. There are both advantages and disadvantages to this approach. Consumption of purified DF allows more specific associations to be made between particular GIT microbial species and the DF being tested, for example, by allowing an examination of bacterial attachment (or not) to specific polysaccharides [177]. However, it may not be a good predictor of the actual behaviour of that fibre within a whole PCW complex, such as those in whole grains, fruits and vegetables [75].

Furthermore, single polysaccharide fibres may be fermented much faster than their incorporated counterparts [178], affecting niche areas of the GIT differently. PCW complexity and structure can slow fermentation by restricting accessibility leading to changes in microbial activity. Previous work has shown that specific bacterial genera are increased by certain DF substrates. For example, an in vitro study of galacturonic acid, the main monosaccharide component of pectin, led to increased *Bifidobacteria* and *Lactobacilli* [179]. Pectin generally is known to pass undigested through the small intestine to the colon [180]. *Lactobacilli* were also increased in a study [181] with sugar beet (high in pectins) and fructooligosaccharides. This found that the addition of these fermentable carbohydrates to a weaning diet of pigs led to a consistent enrichment of *Lactobacilli* in the small intestine.

When pigs were fed commercial inulin, it was reported that the genera *Catenibacterium* and *Blautia* were significantly increased [59]. This was also associated with significantly increased propionic and butyric acid production in the GIT of pigs, compared with the control. Separate work compared inulin with arabinoxylan oligosaccharides [182], and found that inulin was fermented faster and led to microbiota changes more proximally in the LI, while arabinoxylan oligosaccharides were fermented in the distal colon of the LI in vitro model.

Nevertheless, determining specific effects of carbohydrates on the microbiota, either from whole or purified sources, is still very informative, especially when the microbiota is affected so dramatically by the absence of fermentable carbohydrates in the diet [168,183]. In addition, the use of purified DF for these studies is popular due to their potential use as a prebiotic in commercial food products [105].

### 4.3. Whole Plant-Based Food Dietary Fibre and Microbiota

According to Monteiro [76], there are three groups of foods, categorized based on their level of processing as discussed earlier. The first category comprises ingredients which have been minimally processed and includes whole grains, legumes, fruits and vegetables, and it is these which are considered to promote a healthy GIT microbiota [75]. It is this category which will be discussed below. Table 3 presents a summary of mainly in vitro studies using both human and pig inocula, which examined effects of whole plant-based food products on the GIT microbiota diversity.

**Figure 3:**
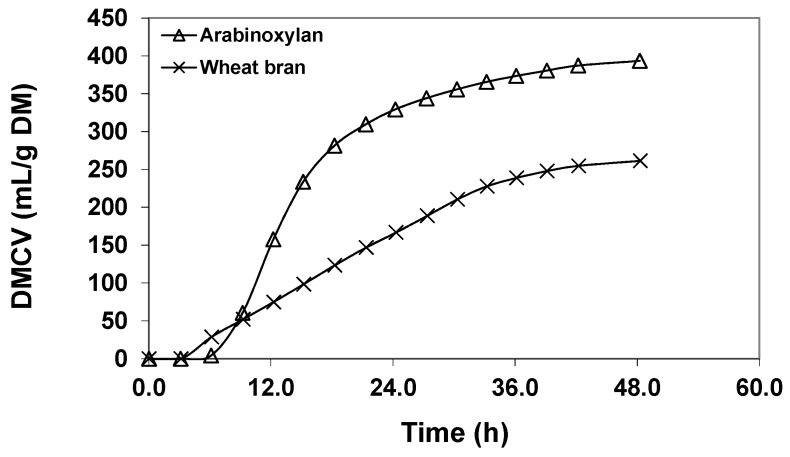
A comparison of in vitro fermentability of arabinoxylan as a pure dietary fibre component versus wheat bran, showing differences in the cumulative gas volumes over time for each substrate, with arabinoxylan readily fermented compared to the more complex wheat bran dietary fibre (Adapted from Williams et al., 2011) [33].

An in vitro study which compared fermentability of purified AX with wheat bran (Figure 3) showed distinct differences in terms of the gas production kinetics in time [33]. The purified material had a much faster rate of gas production, and was fermented to a greater extent, compared with the more complex wheat bran. In broader terms, results such as these have implications for site of fermentation in the GIT, as more slowly fermentable materials will be more likely to ferment for a longer trajectory within the large intestine, compared with a substrate which is fermented more rapidly [112].

### 4.4. Whole Grains

Whole grains commonly refer to all components of the grain (endosperm, aleurone, and pericarp) either intact or in the same proportions as in intact grains from cereal crops such as wheat, rice, barley, maize (cobs), sorghum, oats, and rye [77]. Whole grains can be consumed within bread, or cooked to form dishes to accompany other foods. The nutrient content of whole grains includes fibre, lignans, antioxidant polyphenols, phytosterols, and unsaturated fatty acids [188]. In terms of whole grains versus their processed components, Lappi et al. reported a 37% reduction in Bacteroidetes within the GIT microbiota of human faecal samples, when fed refined wheat bread versus a whole grain rye bread [189]. A similar study by Costabile et al. reported increased amounts of bacteria considered beneficial for those fed a whole grain supplement compared to a more processed wheat bran counterpart [190]. Faecal abundance of *Bifidobacterium* and *Lactobacillus* were significantly increased in the whole-grain diet.

### 4.5. Fruits and Vegetables

Research into the effect of whole food versus purified dietary constituents on GIT microbiota in vivo is very limited, particularly for fruits and vegetables. In one study, Shinohara et al. fed adult humans two apples per day, and concluded that this dietary intervention was associated with a significant increase in *Bifidobacteria* and *Lactobacilli* numbers [186]. In a similar trial looking at bacterial numbers [191], the effect of alfalfa or citrus pulp on GIT fermentation and total bacterial counts was compared with purified inulin, using a pig feeding model. No difference in total bacterial numbers between the three diets were found, and, in an additional in vitro fermentation experiment with a pig faecal inoculum, inulin was found to ferment significantly faster than alfalfa or citrus pulp.

The impact of kiwifruit on the human GIT microbiota, using 454 pyrosequencing was studied in vitro [192]. Despite markedly different baseline diversity of the donor inoculum, kiwifruit increased microbial diversity in vitro. Specifically, increased species within *Bacteroides* and *Bifidobacterium* were found.

The consumption of whole date fruits [193] (7 per day) was compared with no dates in the diet. While this small addition was insufficient to significantly alter the GIT microbiota, it could be argued that the control diet was also balanced in term of added fibre (maltodextrin-dextrose) resulting in similar total fibre content (18.2 g and 18.5 g/100 g for the control and date palm diets respectively). Furthermore, faecal analyses from participants on both diets were significantly enriched with *Bifidobacteria, Lactobacillus, Enterococcus*, and *Bacteroides* genera [193].

## 5. Conclusions

Purified polysaccharide components from DF differ in terms of their effect on the GIT microbial populations and their activity. They are relatively easy to examine in terms of their fermentability, and, due to their comparatively simple structure, may lead to obvious shifts in microbial communities in vitro, and, more importantly, in vivo. Commercial interest is particularly high, due to the possibility of them being added to processed food products with potential claims of prebiotic properties.

Little research has been conducted comparing whole plant-based food products with purified DF polysaccharides extracted from them. One issue here is that extraction of cell walls from plants requires harsh chemical treatments, and then there is considerable discussion of how valid results from this material can be. This has been overcome to some extent by the use of bacterial cellulose composite models, but, even here, these materials are models, rather than actual foods in the human diet. In other words, how specific fibres within the entire PCW may differentially affect the GIT bacterial community, compared with the purified polymer present within that whole food is not implicit. In addition, little is understood about how individual phytonutrients which are abundant in certain fruits and other foods, but adsorbed to a PCW, may affect bacterial communities and their subsequent metabolic outputs compared with the purified compounds.

In addition, while many methods exist to characterise PCW or dietary fibre chemically, it could be argued that, in terms of human nutrition and health, it is the extent and kinetics of fermentability which can have a profound effect on human health in relation to diets containing DF. Therefore, we propose that investigations into techniques which could be used to classify DF according to its fermentability, rather than only chemical definitions, could be a functional approach of immediate relevance to nutrition.

Lastly, there is evidence to indicate that the more complex and varied the diet (and its various plant-based food ingredients), the more complex and varied the resultant GIT microbiota is likely to be. Intuitively, this makes sense, as many bacterial species have the enzymes required for the breakdown of very specific molecules. Hence, the more varied the molecules present, the greater variety of bacteria required to break them down. This field is very much at the beginning stages and will require a thoughtful mix of both in vitro and in vivo techniques to examine the details of three very complex systems simultaneously i.e., human physiology, GIT microbiology, and plant-based food chemistry. Future progress will require close cooperation between microbiologists, plant biologists and food technologists.

## Figures and Tables

**Table 1:** Biological actions of lactic and succinic acids and the predominant short chain fatty acids produced by bacteria in the GIT. Table adapted from [109] with data sourced from [137,138,139,140].

Lactic	Succinic	Acetic	Propionic	Butyric	Mode of Action
		√	√		Source of energy (brain, heart, muscle)
				√	Energy for colonocytes
√		√	√	√	Reduce GIT pH
√		√	√	√	Decreases NH_3_ absorption across epithelium
√		√	√	√	Decreases growth of potential pathogens
				√	Inhibits proliferation and induces apoptosis of cancerous cells
		√	√		Lipid Metabolism
√	√		√		Increased leptin production (increased satiety)
					Involvement in bacterial cross-feeding

**Table 2:** Studies involving various purified DF and the effect on human GIT microbiota.

Purified Ingredient	Methodology Used	Findings	Reference
Polydextrose Soluble maize fibre	454 pyrosequencing of bacterial 16S rRNA genes (V4–V6 region)	Consumption of these fibres led to an increased abundance of faecal Clostridiaceae, Veillonellaceae, Faecalibacterium, Phascolarctobacterium, Dialister and lower Eubacteriaceae	[184]
Maize, Dextrin, Pullulan, Resistant starch (RS)	Micro-array analysis	All tested substrates except RS reduced species of the Bacteroides group, and increased Bifidobacteria	[21]
Aloe vera gel (extract and powder), Larch, *U. pinnatifida* fucoidans, Tragacanth gum, Ghatti gum	Real-time PCR analysis of species of interest	Increased *Bifidobacteria* spp. and the bacteroides-prevotella group	[179]
Amylose, amylopectin, dextran, xylan, polygalacturonate, pectin	Culture analysis of 10 *Bacteroides* spp.	Identified the polysaccharide preference (of the tested substrates) for fermentation by specific bacterial species. Most capable of plant polysaccharide fermentation	[185]
Apple pectin	Faeces were collected for culturing analysis of bacterial populations	Significant increase in *Bifidobacteria* with a decrease in species from the Clostridia class	[186]
High amylase maize starch	Fluorescence in situ hybridisation	*Faecalibacterium prausnitzii* and *Eubacterium hallii* were significantly increased in the cultures	[187]

**Table 3:** Studies involving various whole plant-based food products and the effect on GIT microbiota of humans and animals.

Source of Bacteria	Ingredients	Methodology Used	Findings	Reference
Human faeces	Kiwifruit	In vitro batch culture fermentation, 454 pyrosequencing (V2–V3 region)	*Bacteroides* and *Bifidobacterium* species were more abundant in bacterial communities fermenting kiwifruit	[192]
Human faeces	High flavonoid whole-foods	Total bacterial counts by fluorescence in situ hybridisation	Flavonoid content of whole-foods led to a decreased abundance of potentially pathogenic bacteria, as per relationship to cardiovascular disorders	[166]
Human faeces	Dates	Bacteria enumeration via fluorescent in situ hybridisation	No significant differences of microbiota between diets reported	[193]
Swine in vivo digesta	Alfalfa & citrus pulp	Bacterial culturing for counts	No difference in bacterial counts reported between diets	[191]
Swine faeces	Wheat, wheat bran	Faecal microbiota analysis using qPCR, DNA fingerprinting, metaproteomics	Lactobacilli, bifidobacteria and *Faecalibacterium prausnitzii* was significantly higher (*p* < 0.05) in the high fibre animals. *Enterobacteriaceae* was more abundant in low-fibre-fed animals	[168]
	Ground maize, Wheat bran	Real-time PCR to analyse populations of *Lactobacilli* and *Enterobacteria*	No long-term differences for *Lactobacilli* and *Enterobacteria* between the two diets containing maize and wheat bran. The feeding period of 7 to 42 days showed the enzymatic potential to degrade complex fibres adapted over time. Enzyme activity was detected for xylanase after 7 d and cellulose 42 d	[120]
Swine faeces	Sugar beet pulp, Soybean hulls	Faeces collected for culturing and bacterial counts (log_10_ CFU/g)	Reported that DF did not affect the composition of the bacterial population cultured from faeces	[194]
